# Beyond borders and blockades: human trafficking risks among vulnerable Palestinian populations under occupation

**DOI:** 10.1186/s12889-025-24893-5

**Published:** 2025-10-21

**Authors:** Issam Iyrot, Raid Noairat, Nabila El Meghary

**Affiliations:** 1 Department of Social Sciences, Nablus University for Vocational and Technical Education, Nablus, Palestine; 2 Faculty of Law, An-Najah National University, Nablus, Palestine; 3 Faculty of Legal, Economic and Social Sciences, Sidi Mohamed Ben Abdellah University, Fez, Morocco

**Keywords:** Human trafficking, Palestine, Israeli occupation, Structural vulnerability, Rights-based approach, International law

## Abstract

This article explores the nexus between political occupation, economic marginalization, and the risk of human trafficking in the occupied Palestinian territory (oPt). It argues that the ongoing Israeli occupation and blockade, particularly of Gaza and Area C of the West Bank, have entrenched structural vulnerabilities that increase exposure to exploitation and trafficking, most significantly of women, children, and unregistered workers. The catastrophic escalation following October 7, 2023, has intensified these vulnerabilities to unprecedented levels, creating an acute humanitarian crisis that compounds trafficking risks. Adopting a combined analytical and legal approach, the study utilizes human rights reports, national legislation, and international conventions. The findings indicate that state fragility, fragmented governance, extreme poverty, and the impacts of occupation create fertile ground for labor exploitation, forced displacement, and survival trafficking. The article critiques international anti-trafficking and counterterrorism policies for their decontextualized, criminalized approach, which often ignores the political and colonial histories of occupied lands. It concludes by calling for a rights-oriented, contextualized approach that addresses the structural vulnerabilities arising from occupation and blockade while strengthening national law and international judicial assistance.

## Introduction

Human trafficking, globally defined as “the recruitment, transportation, transfer, harboring or receipt of persons, by means of the threat or use of force or other forms of coercion. for the purpose of exploitation” [1], represents a severe violation of fundamental human rights. Within Palestinian society, the West Bank, the Gaza Strip, East Jerusalem, and refugee camps, this phenomenon is inextricably linked to the prolonged Israeli military occupation and its catastrophic socio-economic impact [2, 3]. The occupation imposes severe restrictions on freedom of movement, access to natural resources, and economic development, fragmenting the territory and creating a context of rampant unemployment, poverty, and social dislocation [4].

The structural vulnerabilities examined in this study, while deeply entrenched over decades of occupation, have reached unprecedented levels following the escalation of conflict since October 7, 2023. The ongoing military operations and complete siege of Gaza have created what the International Committee of the Red Cross describes as a “catastrophic humanitarian situation,” with over 2.3 million Palestinians in Gaza facing acute shortages of food, water, medical supplies, and basic services [5]. The World Health Organization has documented the collapse of Gaza’s healthcare system [6], while OCHA reports that over 1.9 million people, nearly 85% of Gaza’s population, have been internally displaced [4]. These extreme conditions of displacement, family separation, economic collapse, and institutional breakdown create an environment where trafficking risks are exponentially magnified, particularly for women, children, and other vulnerable populations who may resort to increasingly desperate survival strategies.

This paper addresses a pressing concern: How do occupation, blockade, and state weakness drive the heightened risks of human trafficking among vulnerable Palestinian populations? The pivotal hypothesis is that dismissing the occupation’s role leads to an inadequate response, focusing on isolated criminal activities while ignoring the systemic structures that generate vulnerability. This study is guided by two hypotheses:

- H1: The structural circumstances imposed by the Israeli occupation, restricted mobility, economic marginalization, and legal fragmentation, significantly increase the vulnerability of Palestinians, especially women and youth, to various forms of human trafficking.
- H2: The decontextualized nature of international anti-trafficking frameworks perpetuates partial policy efficacy in the Palestinian case by failing to account for the political and colonial determinants of exploitation.

### Conceptual framework

To ground the analysis, this study employs three key concepts:

- Human trafficking: Aligned with the UN Palermo Protocol, trafficking encompasses domestic servitude, sexual exploitation, forced labor, and child recruitment for exploitation [1]. This definition provides a legal baseline for identifying cases but often lacks the contextual depth needed in conflict zones [7].
- Structural vulnerability: This term explains the systemic exposure of individuals or populations to harm due to entrenched social, economic, legal, and political conditions [8]. In the oPt, this vulnerability is a direct consequence of occupation-driven poverty, legal fragmentation, movement restrictions, and institutional weakness [9].
- Structural occupation: Moving beyond mere military control, this concept describes a comprehensive system of domination that includes legal, administrative, economic, and spatial mechanisms designed to exclude self-determination [10]. It entails practices such as land expropriation, resource control, and permit systems that systematically impoverish and disempower the Palestinian population [11].

## Methods

### Study design and data collection

This study applied a qualitative analytical approach combining legal and socio-political analysis. Given the politically sensitive nature of trafficking in the oPt and the challenges of primary data collection due to institutional underreporting and restricted access, a desk-based methodology was deemed most appropriate and ethical [12, 13].

A purposive sampling strategy was employed to select the most relevant reports, legal texts, and academic literature. The data collection timeframe spanned from 2000 (the adoption of the Palermo Protocol) to early 2024. Sources were identified through academic databases (JSTOR, HeinOnline, SAGE Journals) and the online repositories of key international organizations. The selection included:

- International conventions (e.g., Palermo Protocol, International Convention for the Suppression of the Financing of Terrorism).
- UN Security Council resolutions and reports from UN agencies (OCHA, ILO, UNICEF).
- Reports from renowned human rights organizations (Amnesty International, Human Rights Watch, B’Tselem).
- Palestinian national legislation and policy documents.
- Peer-reviewed academic journals focusing on human rights, Middle Eastern studies, and political economy.

### Data analysis

A systematic data extraction matrix was developed to analyze secondary sources methodically. Key variables included: (1) legal safeguards against trafficking and extremism; (2) references to structural weaknesses (occupation, blockade, governance); and (3) documented cases of trafficking and exploitation. Thematic analysis was conducted to identify patterns related to structural vulnerability and institutional response. To ensure validity, findings were triangulated across multiple sources, and interpretations were reviewed independently by both authors, who specialize in international law and Middle Eastern socio-political contexts.

## Results and discussion: structural vulnerabilities in the Palestinian context

The occupied Palestinian territory (oPt) presents a uniquely challenging environment where political, economic, and legal factors converge to create high vulnerabilities to human trafficking. These conditions are not incidental but are fundamentally rooted in the prolonged Israeli military occupation, which imposes a sophisticated regime of restrictions that stifle socio-economic development and basic freedoms [4, 10]. An analysis of these structural circumstances is paramount to understanding the multifaceted nature of trafficking risks.

### Political and mobility constraints

The most dramatic feature of the occupation is the severe limitation on Palestinian mobility. The West Bank is fragmented by a network of Israeli military checkpoints, separation barriers, and closed zones, while Gaza has been subjected to a comprehensive land, air, and sea blockade since 2007 [14]. These restrictions deny Palestinians access to work, healthcare, education, and family, pushing many into vulnerable situations where livelihood options are severely constrained [9].

The Gaza blockade has had a devastating economic impact, leading to crippling shortages of basic commodities, fuel, and building materials. The destruction of infrastructure and the inability to reconstruct have resulted in extreme poverty and dependency on humanitarian aid. According to the International Labour Organization, unemployment rates in Gaza are among the world’s highest, with youth unemployment exceeding 60% [15]. This economic stranglehold pushes many Gazans into precarious informal labor markets where the risk of exploitation is high and protections are minimal [16].

In Area C of the West Bank, which comprises 60% of the area and is under full Israeli control, restrictions on Palestinian development and land use severely limit economic opportunities. Palestinians are often displaced or forced into low-wage, informal work, both domestically and in Israel, where they face exploitative conditions including wage theft and hazardous environments [3].

### Economic vulnerability and social fragmentation

Economic marginalization is exacerbated by domestic political fragmentation between the Palestinian Authority (PA) in parts of the West Bank and Hamas in Gaza. Women and youth bear the brunt of this economic weakness. Deeply rooted patriarchal structures, combined with limited formal job opportunities, ensnare women in vulnerable informal sectors or domestic service, which are characterized by low pay, a lack of contracts, and a high risk of abuse [17, 18]. Young Palestinians, facing high unemployment and limited social mobility, are exposed to false recruitment promises for work or migration that can easily devolve into trafficking [15].

Refugee and internally displaced populations face compounded risks. Often living in congested, unhygienic conditions with limited access to education and healthcare, they are highly susceptible to abuse. A lack of social services, protection structures, and fear of stigma within traditional communities further discourages reporting and access to assistance [2, 19].

### Fragmented legal and institutional frameworks

The legal and institutional landscape in the oPt is characterized by profound fragmentation. Overlapping jurisdictions, Israeli military law in Area C and settlements, Palestinian civil law in Areas A and B, and various emergency regulations, create a complex system with poorly articulated obligations and significant enforcement gaps [20]. For instance, the PA has no authority over cases involving Palestinians working in Israel or settlements, severely limiting judicial recourse for victims of trafficking or exploitation [21].

The PA’s capacity to enforce its own anti-trafficking laws is weak, hampered by a lack of resources, inadequate training for judicial officers, and political instability. Victim protection services, such as shelters, rehabilitation, and psychosocial support, are limited and heavily reliant on under-resourced civil society organizations and international funding [22, 23]. This institutional weakness leads to significant underreporting, as victims fear stigma, retaliation, or simply do not trust the authorities [24].

### Populations at heightened risk

The intersection of economic marginalization, social exclusion, and political limitations creates a fertile environment for exploitation, rendering specific groups extremely susceptible to trafficking networks.

#### 1. Women and girls

Palestinian women, particularly in Gaza and poorer areas of the West Bank, face acute social and economic pressures. The United Nations Population Fund reported a dramatic increase in early and forced marriages in Gaza, often linked to economic desperation and societal insecurity [25]. Such marriages can constitute trafficking for sexual exploitation or domestic servitude, yet cultural shame often silences victims, preventing access to justice. Economic exclusion forces women into survival schemes or informal home-based employment; female labor force participation was just 15% in 2020, indicating both limited opportunities and social constraints [17].

#### 2. Youth and children

Youth unemployment is a perpetual crisis. The ILO reported Gaza’s youth unemployment rate at 63% and the West Bank’s at over 40% [15]. Consequently, many young Palestinians resort to irregular migration channels, often facilitated by trafficking networks promising employment abroad. Child labor remains widespread, especially in agriculture and informal urban markets. UNICEF documented cases of children as young as 12 engaged in hazardous labor, with impunity from the law and no access to schooling [26]. Inadequate enforcement mechanisms embolden such exploitation.

#### 3. Migrant workers and refugees

Palestinian workers in Israel and settlements are exposed to highly exploitative conditions. A report by the Palestinian Human Rights Organizations Council documented widespread wage theft, a lack of contracts, and dangerous working conditions for these workers, whose legal status largely prevents them from seeking remedies [23]. Palestinian refugees in neighboring countries like Jordan, Lebanon, and Syria are also vulnerable. The International Organization for Migration noted that refugees are soft targets for trafficking under the guise of labor recruitment or fake migration opportunities, which often result in forced labor [27].

#### 4. Internally displaced persons (IDPs)

Ongoing home demolitions in Area C and conflicts in Gaza have internally displaced thousands. IDP families lose access to community protection and livelihoods. A UNRWA report highlighted that IDPs in West Bank camps are at greater risk of trafficking due to their vulnerable living conditions and lack of legal protections [19].

##### Integrated case evidence

The vulnerability of these groups is not theoretical. For example, in 2021, a Palestinian teenage girl from Gaza was trafficked through a fake recruitment scheme for domestic service in the Gulf, ending up in a situation of forced labor and ill-treatment [28]. Furthermore, as of June 2023, approximately 164,000 Palestinians were employed in Israel and settlements, primarily in construction, agriculture, and industry, with most facing exploitative labor conditions due to inadequate legal protection [21]. Data from 2018 revealed that 4,840 children in Gaza aged 10–17 worked full-time, with another 1,490 working part-time while studying, representing approximately 2% of that age group [29]. This data, though fragmented, paints a clear picture of systemic vulnerability directly tied to the political economy of occupation.

### A critical review of the global anti-trafficking framework

International efforts to combat human trafficking have been primarily shaped by multilateral frameworks such as the UN Palermo Protocol [1], complemented by regional initiatives and national reporting mechanisms. These frameworks have raised global awareness and standardized definitions, yet when applied to complex, politically charged contexts like Palestine, they exhibit grave shortcomings that merit critical examination [7, 30].

### The broader framework: beyond the TIP report

While the U.S. Department of State’s Trafficking in Persons (TIP) Report represents one of the most prominent evaluation mechanisms within the global anti-trafficking architecture, it operates within a broader ecosystem of international frameworks that share similar conceptual limitations. The global anti-trafficking framework encompasses multiple interconnected components:

- The UN Palermo Protocol serves as the foundational legal instrument, establishing the internationally accepted definition of trafficking and the “4P” approach (Prevention, Protection, Prosecution, and Partnerships). However, this framework’s emphasis on criminal law responses often overshadows the structural drivers of vulnerability, particularly in contexts of political conflict and occupation.
- Regional mechanisms such as the Council of Europe Convention on Action against Trafficking in Human Beings and the ASEAN Convention Against Trafficking in Persons similarly prioritize law enforcement cooperation and victim protection services, while maintaining limited engagement with the political economies that generate trafficking vulnerabilities.
- International monitoring bodies, including the UN Office on Drugs and Crime (UNODC) and various UN Special Rapporteurs, conduct assessments using standardized metrics that often fail to account for the unique challenges faced by occupied or conflict-affected territories.
- The U.S. TIP Report, while influential due to its potential sanctions implications, is representative of this broader framework’s methodological approach and conceptual limitations. Its annual country assessments reflect the dominant paradigm’s emphasis on state capacity, law enforcement statistics, and victim services—criteria that assume functional sovereignty and institutional autonomy.

### Systemic decontextualization across multiple frameworks

The decontextualization critique applies broadly across these various mechanisms. The primary limitation is their collective failure to adequately integrate political economy analysis into trafficking assessments. This manifests in several ways:

- Methodological standardization: Most frameworks apply uniform evaluation criteria across diverse political contexts, failing to differentiate between sovereign states, occupied territories, and conflict zones. This standardization obscures how occupation, colonialism, and structural violence create unique vulnerabilities that require contextualized responses.
- State-Centric focus: The frameworks assume state responsibility and capacity while inadequately accounting for situations where state institutions are constrained, fragmented, or operating under external control. In Palestine’s case, this results in assessments that critique Palestinian institutional performance without acknowledging the structural limitations imposed by occupation.
- Criminalization emphasis: Across different mechanisms, there remains a persistent focus on prosecution statistics and law enforcement capacity as primary indicators of anti-trafficking effectiveness. This approach marginalizes prevention strategies that address root causes, particularly those related to political oppression and economic marginalization.
- Insufficient political analysis: The frameworks generally treat trafficking as a criminal phenomenon rather than as an expression of deeper political and economic injustices. This depoliticization limits their capacity to address trafficking in contexts where political factors are primary drivers of vulnerability.

### The TIP report as exemplar, not outlier

The U.S. TIP Report’s treatment of Palestine exemplifies these broader systemic issues rather than representing a unique analytical failure. Its 2024 assessment of Palestinian anti-trafficking efforts—criticizing insufficient prosecutions, inadequate victim identification, and limited protection services while minimally acknowledging occupation-related constraints—reflects the methodological limitations inherent across the global framework.

This assessment treats trafficking in Palestine as comparable to trafficking in sovereign states, applying benchmarks that assume functional borders, unified legal systems, and autonomous institutional authority. The report’s superficial acknowledgment that “Israeli authorities’ restrictions on Palestinian movement and economic activity contributed to Palestinians’ vulnerability to forced labor” demonstrates the tokenistic manner in which structural factors are incorporated across various international mechanisms.

### Toward structural and rights-based reframing

This systemic analysis necessitates a fundamental reorientation of anti-trafficking strategies across multiple levels. Scholars and activists increasingly advocate for integrating political economy analysis and human rights principles throughout the global framework [7]. This reorientation would:

- Situate trafficking not solely as a crime but as an expression of deeper systemic violations and political repression.
- Develop context-specific evaluation criteria that account for political constraints on institutional capacity.
- Prioritize structural prevention strategies that address root causes rather than reactive enforcement measures.
- Integrate demands for political justice, including the removal of occupation-related barriers, into anti-trafficking advocacy.

In occupied contexts like Palestine, this transformation means understanding how military occupation, legal fragmentation, and colonial practices create vulnerability, and shifting from punitive enforcement toward protection, empowerment, and structural reform [11].

### Local gaps and legal responses in Palestine

The Palestinian territories face profound difficulties in combating human trafficking due to the nascent and vulnerable nature of their legal and institutional infrastructures, compounded by political and territorial fragmentation.

### Legislative and enforcement challenges

Although the Palestinian Authority has enacted laws criminalizing human trafficking, most notably Presidential Decree No. 16 of 2014 on Combating Human Trafficking, which aligns Palestinian legislation with the UN Palermo Protocol and criminalizes all forms of trafficking in persons, enforcement is severely lacking [20]. This decree established penalties of 7–15 years imprisonment for trafficking offenses and created institutional frameworks for victim protection, yet its implementation remains inadequate due to structural constraints. Significant challenges include:

- Limited Institutional Capacity: Police, judicial, and prosecutorial institutions lack specialized training, resources, and infrastructure to effectively investigate and prosecute complex trafficking cases.
- Political Instability: The chronic political division between the West Bank and Gaza hampers consistent policy implementation and collective action.
- Corruption and Impunity: Widespread corruption and a culture of impunity undermine trust in institutions and deter victims from seeking justice.
- Ineffective Victim Identification: Law enforcement officials are often untrained in identifying victims of trafficking, leading to survivors being misclassified as criminals, irregular migrants, or illegal workers [21].

### Gaps in victim protection and support

Protection for trafficking survivors in Palestine is weak. There are few specialized shelters or rehabilitation centers, and existing facilities are overcrowded and lack essential services like psychological counseling, legal aid, and medical care. Social stigma, particularly for women and girls subjected to sexual exploitation, silences victims who fear isolation, family rejection, or retaliation from traffickers, leading to extreme underreporting [2].

International and local NGOs, such as the Palestinian Women’s Union and the Ma’an Network, provide critical services but operate under significant constraints, including limited funding, movement restrictions due to occupation policies, and sporadic political interference. Consequently, the scale and quality of victim services remain insufficient to meet the need [31].

## Conclusion and recommendations

This analysis has demonstrated that human trafficking in the oPt is a multifaceted phenomenon qualitatively shaped by the specific political, economic, and legal conditions of prolonged occupation. The structural violence, economic marginalization, and legal fragmentation inherent in the occupation context are the primary drivers of vulnerability. The catastrophic humanitarian crisis following October 7, 2023, has intensified these vulnerabilities to unprecedented levels, with massive displacement, family separation, and institutional collapse creating conditions where trafficking risks are exponentially magnified. International anti-trafficking regimes, while useful, often fail to capture these underlying determinants, promoting decontextualized and criminalized responses that are insufficient.

A paradigm shift is urgently needed, from reactive enforcement towards comprehensive, justice-based approaches. Based on our findings, we propose the following integrated recommendations:

1. Contextualize Anti-Trafficking Strategies: Donors and policymakers must adopt context-specific models that integrate political economy analysis. Strategies must address systemic barriers like movement controls, economic deprivation from the blockade, and legal fragmentation, aligning anti-trafficking efforts with broader human rights and development objectives. In the current crisis, this includes addressing the acute vulnerabilities created by mass displacement and institutional breakdown.
2. Consolidate Legal and Institutional Frameworks: Legislative reform must prioritize creating a unified legal system applicable across all Palestinian territories. Capacity-building for police, prosecutors, and judges in victim identification, trauma-informed care, and prosecution techniques is essential. Inter-agency coordination mechanisms must be established.
3. Empower Vulnerable Populations: Prevention must focus on the economic and social empowerment of high-risk groups through education, vocational training, and sustainable livelihoods. Support services must be significantly expanded to include safe shelters, comprehensive psychosocial counseling, legal assistance, and community awareness campaigns to combat stigma.
4. Strengthen Data Collection and Research: A coordinated, nationwide data collection system involving government, civil society, and international actors is crucial for evidence-based policy. Supporting independent qualitative and quantitative research on trafficking dynamics is needed to inform targeted interventions.
5. Foster Regional and International Solidarity: Given the transnational nature of trafficking and Palestine’s unique geopolitics, collective action is necessary. This includes strengthening cross-border coordination with neighboring states and engaging international actors to advocate for the removal of occupation-related barriers that exacerbate trafficking risks.

Only through such a comprehensive, context-sensitive approach that addresses both immediate protection needs and underlying structural injustices can meaningful progress be made toward protecting the dignity and rights of all Palestinians.

## Data Availability

The datasets generated and analyzed during this study are included in the published article or are available from the corresponding author upon reasonable request.
